# Non-invasive EEG-based BCI spellers from the beginning to today: a mini-review

**DOI:** 10.3389/fnhum.2023.1216648

**Published:** 2023-08-23

**Authors:** Olga Maslova, Yuliya Komarova, Natalia Shusharina, Alexander Kolsanov, Alexander Zakharov, Evgenia Garina, Vasiliy Pyatin

**Affiliations:** ^1^Neurosciences Research Institute, Samara State Medical University, Samara, Russia; ^2^Baltic Center for Neurotechnologies and Artificial Intelligence, Immanuel Kant Baltic Federal University, Kaliningrad, Russia; ^3^Department of Operative Surgery and Clinical Anatomy with a Course of Innovative Technologies, Samara State Medical University, Samara, Russia; ^4^Department of Physical Culture, I.M. Sechenov First Moscow State Medical University (Sechenov University), Moscow, Russia

**Keywords:** brain-computer interface, people with disabilities, motor imagery, P300, speller

## Abstract

The defeat of the central motor neuron leads to the motor disorders. Patients lose the ability to control voluntary muscles, for example, of the upper limbs, which introduces a fundamental dissonance in the possibility of daily use of a computer or smartphone. As a result, the patients lose the ability to communicate with other people. The article presents the most popular paradigms used in the brain-computer-interface speller system and designed for typing by people with severe forms of the movement disorders. Brain-computer interfaces (BCIs) have emerged as a promising technology for individuals with communication impairments. BCI-spellers are systems that enable users to spell words by selecting letters on a computer screen using their brain activity. There are three main types of BCI-spellers: P300, motor imagery (MI), and steady-state visual evoked potential (SSVEP). However, each type has its own limitations, which has led to the development of hybrid BCI-spellers that combine the strengths of multiple types. Hybrid BCI-spellers can improve accuracy and reduce the training period required for users to become proficient. Overall, hybrid BCI-spellers have the potential to improve communication for individuals with impairments by combining the strengths of multiple types of BCI-spellers. In conclusion, BCI-spellers are a promising technology for individuals with communication impairments. P300, MI, and SSVEP are the three main types of BCI-spellers, each with their own advantages and limitations. Further research is needed to improve the accuracy and usability of BCI-spellers and to explore their potential applications in other areas such as gaming and virtual reality.

## 1. Introduction

The natural human ability to interact with other people in the form of joint activity, verbally or with gestures depends on the functional status of the cognitive and neuromuscular systems. Most often, this natural human ability became the problem when there is a complete or partial loss of control over the muscle actions. Hard loss of the muscle control is damage to the central motor neuron unites a group of neurological diseases, among which the most well-known chronic disease is the amyotrophic lateral sclerosis, which is based on damage to the motor neurons of brain and spinal cord (Zakharov et al., 2020; Verbaarschot et al., 2021). It is well known that a similar course of neurological disorders is observed in brainstem stroke, locked-in syndrome, brain or spinal cord trauma, cerebral palsy, multiple sclerosis, and muscular dystrophies, the progression of which leads to the fact that patients lose the ability to control voluntary muscles, thereby causing functional and cognitive disorders (Rezeika et al., 2018). People who have lost their motor functions lose the ability to communicate with other people, which in turn leads to an increasing in frustration, depression and, as a result, to social deprivation.

Two decades ago, scientists proposed a new technological paradigm for patients who had lost control of their muscles, namely the Brain-Computer Interface (BCI) paradigm. BCI provides patients with motor diseases the opportunity to communicate using, for an example, the cortex electrical signals. There are many methods to monitor the brain activity in the BCI paradigm: Electroencephalogram (EEG), as well as Electrocorticography (ECoG), functional Magnetic Resonance Imaging (fMRI), and Positron Emission Tomography (PET). Between it all the EEG method is a non-invasive measurement technique widely used in almost all modern BCI applications, more practical than which requires an opening through the skull to directly access the brain tissue. The main reasons why EEG is so common are follows: EEG equipment is relatively inexpensive, portable, simple to set-up, and provides a signal with high time resolution compared to other non-invasive methods for monitoring brain activity. Also, non-invasive BCIs could become a useful tool to be utilized and tested by healthy individuals for research and development of applications (Wolpaw et al., 2002). The BCI-speller is one of the first software applications that kick-started many advances in the brain-to-computer communication. Spelling is one of the first BCI application, it corresponds to the main communication mean for people who are unable to speak (Cecotti, 2011). A variety of BCI spellers has been designed and tested. The most well-known one is the visual P300 speller, which detects the brain response to an attended oddball stimulus (Farwell and Donchin, 1988).

To date, the BCI and speller paradigms have been described in sufficient detail in the scientific literature, starting from the first publications on models of these paradigms (Farwell and Donchin, 1988; Wolpaw et al., 2002; Rezeika et al., 2018). As a results three major Brain–Computer Interface (BCI) paradigms are known now: P300 paradigm, Steady-State Visual Evoked Potential (SSVEP) paradigm, Motor imagery (MI) paradigm. P300 or the oddball paradigm causes a P300 signal in the brain waves of the user which is then interpreted by the BCI system, resulting in the selection of the desired letter. Researchers have developed both visual and auditory stimuli to induce a P300 signal for different systems and applications: Visual Evoked Potential (VEP), Auditory Evoked Potential (AEP), tactile P300 BCI (Farwell and Donchin, 1988; Höhne and Tangermann, 2014; Bulanov et al., 2020a). The SSVEP system is implemented in several modifications: a frequency-modulated Visual Evoked Potential (f-VEP); the code-modulated Visual Evoked Potential (c-VEP); the Motion-Onset Visual Evoked Potential (mVEP) (Snyder, 1992; Guo et al., 2008; Bulanov et al., 2020b). In particular, the mVEP paradigm has been used to investigate human brain motion processing, and mVEP (pre-defined simple motion of the visual targets) comprised of the peaks P1, N2 (160–200 ms), and P2 (complex visual moving stimuli, latency 240 ms). Analysis of publications in this subject area showed that both BCI and Speller paradigms are undergoing constant evolution. New approaches to the development of the BCI-speller paradigm are aimed at achieving highly effective communication of patients or healthy subjects between each other or with external devices (Sun et al., 2022).

## 2. Paradigms of spellers: BCI-P300, BCI-MI and BCI-SSVEP

### 2.1. BCI–P300 paradigm

The first BCI-speller application on the P300 wave was introduced by Farwell and Donchin (1988), consisting of a 6 × 6 matrix illuminated randomly (Farwell and Donchin, 1988). Since the matrix consisted of six rows and six columns, at least 12 flashes were required for each column and row to blink once. The subject focused on the target letter and counted the number of flashes of the required symbol/letter for the better concentration of attention. The brighter illumination of the row and column containing the target symbol as a visual stimulus caused the P300 wave in the EEG signals. The maximum achieved precision was 95% at 12 bits/min, so the required symbol was classified by the computer from the matrix after about 26 s in Farwell and Donchin (1988). It was a very long time for typing compared to the abilities of a healthy person, but was of great importance to a person who was not available any other ways of communicating. Thus, it was proved that the P300 could be used to develop BCI communication applications. Technology has evolved over the years toward faster, more accurate, and user-friendly BCI P300-speller.

**FIGURE 1:**
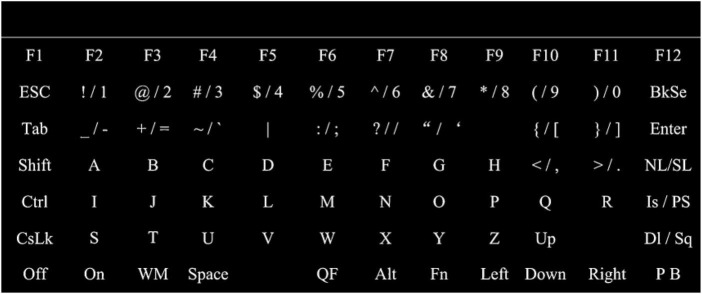
Example of the keyboard type matrix and the stimulus screen presented to the subject during the experiment.

Thus, Jin et al. (2010) reduced the number of highlights per attempt from the classic variant of 12 flashes, which was mentioned above, to 9 and 7, which speeded up the application. Nine lights showed the highest accuracy and 92.9% information rate at 14.8 bits/min, and 12 flashes showed 88.0% and 10.1 bits/min, respectively, while 7 lights showed 68.8% and 5.3 bits/min. The maximum rate was 17.3 bits/min, but with a lower accuracy of 89.3% for 9 flashes per attempt. In addition, Jin et al. (2012) proposed a 7 × 12 keyboard type matrix. Such solution improved the classification accuracy of P300 up to 94.8% at a speed of 27.1 bits/min for 21 flash templates (Figure 1).

**FIGURE 2:**
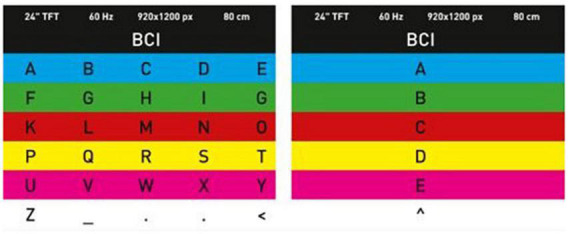
Example of the screen with visual stimulus of different colors presented to the subject during the experiment.

Chroma Speller developed by Acqualagna et al. (2013) provided visual stimuli of different colors. The subjects were asked to focus not on the letter, but on the color. The Chroma Speller was created as a system that was independent of sight, and that required minimal workload. The research showed high level of productivity (Figure 2).

**FIGURE 3:**
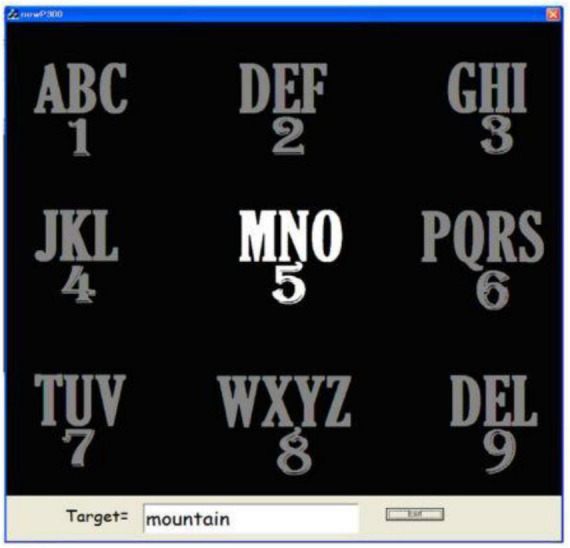
Example of the matrix interface and the stimulus screen presented to the subject during the experiment.

Akram et al. (2015) used a 3 × 3 matrix interface (Figure 3) to reduce the typing time, which is similar to a smartphone keyboard with a built-in dictionary that suggests words as the user types the characters he needs, similar to the predictive typing system T9 (Text on nine keys) on the mobile phones. As a result, it was possible to achieve a reduction in word typing time from 3.47 min for the traditional BCI-speller to 1.67 min in the proposed paradigm, which in turn reduced the word typing time by 51.87%.

**FIGURE 4:**
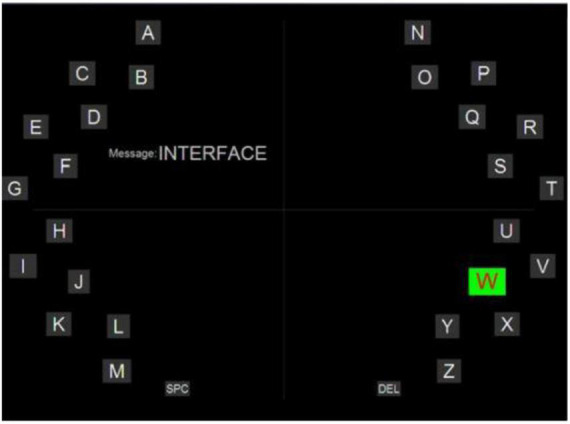
Example of the side single-character speller module based on the P300.

Pires et al. (2012) created a side single-character speller module based on the P300. The productivity of the lateral single-character writing was compared with the productivity of standard spelling–rows and columns. The results showed that the accuracy of the technology was 89.90% at a speed of 26.11 bits/min, and 88.36% at a speed of 21.91 bits/min for rows and columns (Figure 4).

**FIGURE 5:**
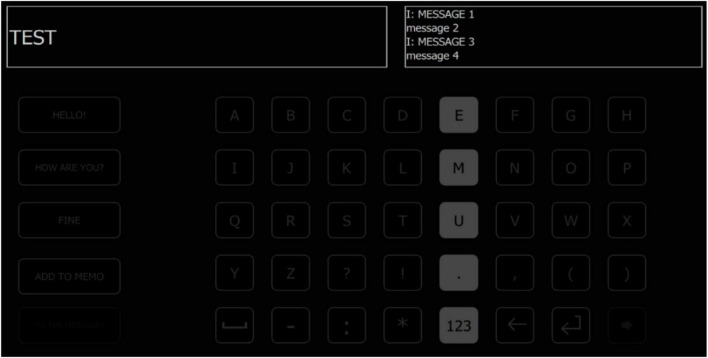
Example of the “Neurochat” interface.

Relatively recently, on the basis of the BCI-P300, Russian developers have created a software and hardware complex “Neurochat” which were presented by the convenient user interface (Figure 5), special neuroheadset and Wi-Fi method of connection a headset to a computer. This system allows people with severe speech disorders or a lack of hand motor skills to communicate on the Internet. Studies of typing by patients with post-stroke aphasia using “Neurochat” have shown a progressive increasing in the accuracy of typing words from 63% in the first session to 92% in the tenth session (Ganin et al., 2020).

**FIGURE 6:**
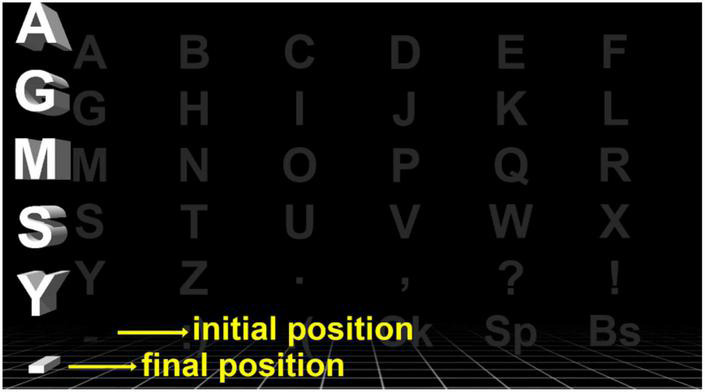
Example of the ex-three-dimensional visualization and column highlighting.

Unlike classical BCI spellers based on the two-dimensional approach of flashing rows and columns, Korkmaz et al. (2022) proposed using a three-dimensional visualization and column highlighting (Figure 6). This idea was based on the facts that, firstly, the P300 wave amplitude when using columns is greater than when using rows due to people’s habit of reading texts horizontally, and secondly, 3D visualization increases the brain’s ERP response. Significance testing is also used to evaluate performance improvement, and it was noted that the proposed paradigm significantly improves performance with fewer electrodes. When using the proposed paradigm, the best mean classification accuracy scores on test data improve from 89.97 to 93.90% (an improvement of 4.36%) for 1 flash, from 97.11 to 98.10% (an improvement of 1.01%) for 3 flashes, and from 99.70 to 99.81% (an improvement of 0.11%) for 15 flashes when using all electrodes included in the study. On the other hand, accuracy scores increase by 9.69% for 1 flash, 4.72% for 3 flashes, and 1.73% for 15 flashes when using the proposed paradigm with only one EEG electrode (P8).

### 2.2. BCI—MI paradigm

**FIGURE 7:**
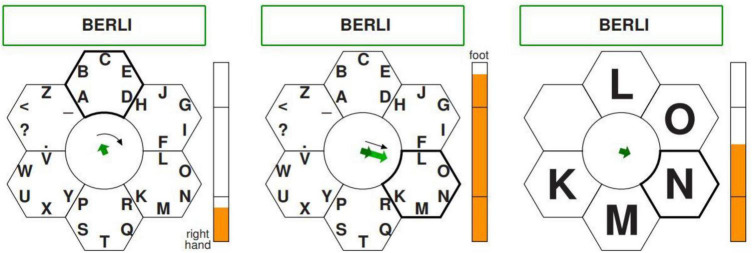
Example of the stimulus screen presented to the subject during the experiment by the Hex-o-Spell system.

The first BCI-speller system based on the motor images (MI) was introduced in 2006 and called Hex-o-Spell (Blankertz et al., 2007). The developers were inspired by the Hex system, which was used in mobile phones and allowed to type text by changing the orientation of the device. The BCI system was represented by two controls: an imaginary movement of the right hand and an imaginary movement of the leg for 30 purposes, consisting of 26 letters of the English alphabet and 4 punctuation marks (Figure 7).

The graphical interface was represented by six hexagons arranged around a circle, in the center of which there was an arrow pointing to any of the hexagons, each of which contained five characters. Imagining the movement of the right hand or foot, the user could rotate the arrow and select the hexagon in which the target letter was located. After that, the selected group of symbols was arranged in such way that each symbol occupied one of the hexagons, while an empty hexagon made it possible to return to the previous stage in case of an error. The typing speed in the MI-based IMC system varied between 2.3 and 5 symbols per minute for one user and from 4.6 to 7.6 symbols per minute for another user. In general, the system had the advantages of MI: independence from the gaze and the absence of the necessary stimulation, but long-term training in the reception of motor imagination was required from users. The BCI method on the motor imagery paradigm is also evolving. The sensorimotor rhythm can be recorded over the motor cortex with the contribution of some somatosensory areas. Based on sensorimotor rhythm EEG BCI-MI is used to improve the motor acts in healthy people and to restore the neuromuscular system in patients.

Classically during the movement in Motor Imagery (MI) and movement preparation the sensorimotor rhythm demonstrates the Event-Related Desynchronization (ERD) and Event-Related Synchronization (ERS). Wherein the signal location varies depending on which limb is moving and on which side of the body the specific movement is taking place. Despite the fact that during MI the amplitude of the sensorimotor signals is not large, the classification makes it possible to distinguish between the imagination of the leg or arm movement, as well as the side of the upper or lower limb (left or right) (Wolpaw, 2013). In the study (Varsehi and Firoozabadi, 2021), the increase in the reliability of the BCI-MI method is based on the remarkable idea–to ensure the selection of the minimum number of the EEG signal channels, which significantly improves the quality of classification of the brain signals generated during motor imagery. This is done by the Granger cause-and-effect analysis, as well as using the machine learning method, independent component analysis of clusters of artifactual and normal EEG signals. After selecting a small number of EEG channels, BCI-MI achieves a classification accuracy of 93.03%, sensitivity of 92.93%, and specificity of 93.12%.

### 2.3. BCI—SSVEP paradigm

**FIGURE 8:**
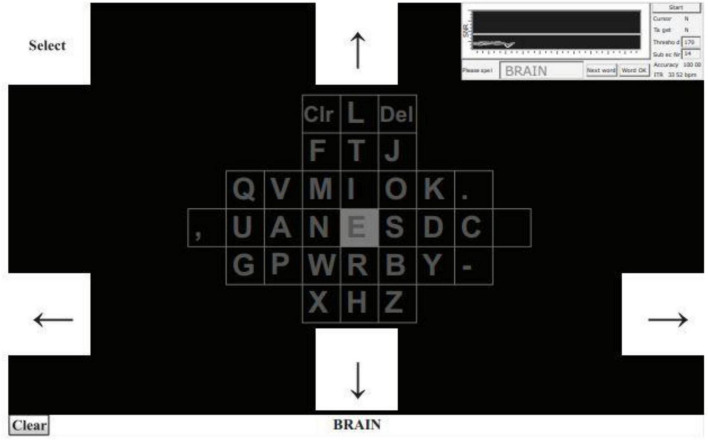
Example of the rhombus-shaped keyboard.

Spelling is one of the first BCI application. It corresponds to the main communication mean for people who are unable to speak (Wolpaw et al., 2002). BCI-speller SSVEP, based on Steady-State Visual Evoked potentials (SSVEP), does not require calibration or special training for the user (Volosyak et al., 2009). The technology was represented by a rhombus-shaped keyboard and contained 26 letters of the English alphabet, 3 punctuation marks, and the “Ctrl” and “Del” keys (Figure 8). Four fields with arrows located on the periphery of the keyboard made it possible to move the keyboard cursor left and right, up and down, and the “Select” field was designed to select the target character. Each of these fields flickered at a certain frequency, causing SSVER in the cerebral cortex. In addition, the technology reproduced the name of the letter selected by a user. Studies have shown that the classification accuracy of SSVER at an average information transfer rate of 22.6 bits/min was 92%.

Among other things, various devices were added to speller systems based on the P300, MI, and SSVEP paradigms to speed up the BCI-speller and improve classification accuracy, for example, an eye tracker or combine paradigms, for example, P300 with MI or P300 with SSVEP (Yin et al., 2014; Mannan et al., 2020; Zuo et al., 2020). A new version of the BCI-speller with a reduced visual field, developed and studied by an inter-university group of scientists, showed a higher classification accuracy than the classic BCI-P300 (Kirasirova et al., 2020). A study showed that BCI with 120 targets based on code-modulated visual evoked potentials (c-VEP) has a higher average information rate (265.74 bit/per min), the large instrument set, high speed, and short training time simultaneously (Sun et al., 2022). The instrument set can be further expanded by enlarging the code combination. At the same time, the average accuracy achieved of 76.58%. Although the brain-computer interface (BCI) based on a stationary visual evoked potential (SSVEP) has been widely studied due to its high information transfer rate, short user training time, and wide subject applicability. However, this method also has disadvantages such as visual discomfort and “BCI illiteracy.”

In the study to create a hybrid BCI (h-BCI) system, the authors used low-frequency stimulation (12 sessions, 0.8–2.12 Hz with an interval of 0.12 Hz), which evokes visual evoked potential (f-VEP) and pupillary reflex (PR) (Jiang et al., 2022). The final hybrid accuracy was obtained using a decision fusion method to combine VEP and PR information. As a result, the average accuracy was 94.90 ± 2.34% (data frame 1.5 s) for the controlled method and 91.88 ± 3.68% (data frame 4 s) for the unsupervised method, which corresponds to an information transfer rate of 64.35 ± 3.07 bits/min and 33.19 ± 2.38 bits/min, respectively. The authors conclude that the proposed h-BCI with the low-frequency visual stimulation paradigm is more convenient and beneficial than the traditional SSVEP-BCI paradigm using the alpha frequency band.

## 3. Speller—Hybrid systems headings

Hybrid systems were designed to combine the advantages of two different systems.

**FIGURE 9:**
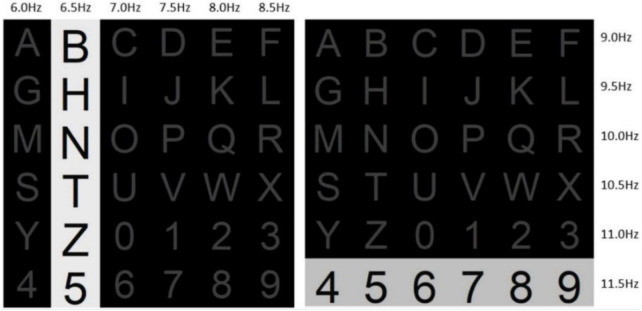
Example of the hybrid speller in the frequency enhanced row and column (FERC).

Bai et al. (2023) proposed a hybrid speller in the frequency enhanced row and column (FERC) paradigm, which allows for the simultaneous elicitation of two signals, P300 and SSVEP. The graphical interface used was a classic 6 × 6 row-column layout, equal to 36 characters (Figure 9). It is worth noting that the frequency of column flashing was 6.0, 6.5, 7.0, 7.5, 8.0, and 8.5 Hz, and the frequency of rows was 9.0, 9.5, 10.0, 10.5, 11.0, and 11.5 Hz, with each row or column flashing for 1 s. Thus, as soon as the classifier recognizes the column and row, the target stimulus is determined. The proposed hybrid BCI achieved an average accuracy of 94.29% and an information transfer rate of 28.64 bits/min.

Another hybrid BCI-speller system (Han et al., 2023), encoded by a combination of electroencephalography functions: P300, motion visual evoked potential (mVEP), and SSVEP, used a layout of more than 200 targets instead of the common 36 characters. The authors noted that the mVEP and P300 components were prominently expressed in the central, temporal, and occipital regions, while the most distinct feature of SSVEP was observed in the occipital region. The proposed hybrid BCI-speller achieved an average accuracy of 85.37 ± 7.49% and 86.00 ± 5.98% for the classification of 216 targets, with an average information transfer rate of 302.83 ± 39.20 bits/min and 204.47 ± 37.56 bits/min, respectively.

**FIGURE 10:**
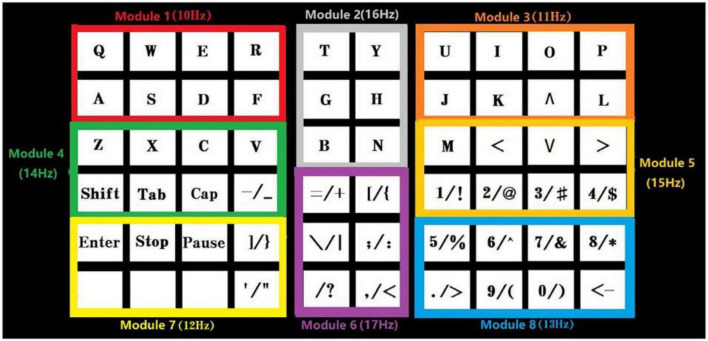
Example of the brain-controlled BCI-speller with an EOG and SSVEP-based.

Zhang Z. et al. (2023) developed a brain-controlled BCI-speller with an EOG and SSVEP-based switch that allows for the activation and deactivation of stimulus illumination in a waiting state, reducing visual fatigue. EOG represents the depolarization and hyperpolarization between the cornea and retina caused by eye movement, which creates a potential difference between the retina and cornea. The distinctive feature of EOG from EEG is that its amplitude is higher than background physiological signals, making it easier to detect. The results showed that the accuracy of the brain-controlled switch developed in this study was up to 94.64%. Additionally, the study design itself is based on the time-space frequency conversion (TSFC) and SSVEP paradigm (Figure 10), which operates on the constant change of the temporal and spatial frequency of auxiliary SSVEP stimulus blocks. The developed 60-character orthography, based on the TSFC-SSVEP stimulus paradigm, has an accuracy of 90.18% and an information transfer rate of 117.05 bits/min.

**FIGURE 11:**
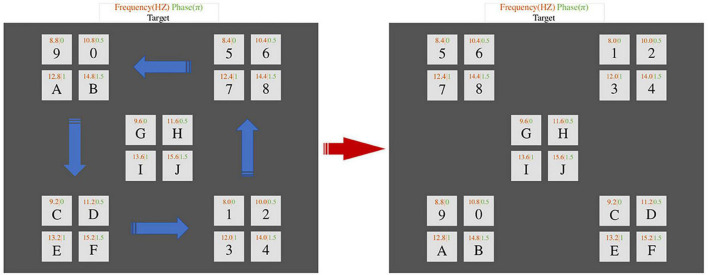
Example of the hybrid BCI combining SSVEP and EOG-based eye movements.

At the same time, Zhang J. et al. (2023) also proposed combining SSVEP and EOG to improve the performance of the BCI-speller. The target stimulus selection process occurs as follows: in the first stage, 20 buttons start flashing simultaneously, and the subject selects the desired symbol and looks at it. Then, eye movement stimulation occurs, meaning that when the sinusoidal stimulation (first stage) ends, 16 buttons in four areas of the graphical interface start moving in different directions. The user continues to track the target with their eyes, making corresponding eye movements. The Figure 11 shows how the speller’s graphical interface looks from the beginning of button movement to the end of movement. The conducted experiment demonstrated an average system accuracy and information transfer rate of 94.75% and 108.63 bits/min, respectively.

### 3.1. SSVEP + EMG

Lin et al. (2016) created the orthographic matrix 6/10. Sixty symbols were divided into four groups of 15 characters, which were illuminated with different frequencies. The participant made the first movement for the group selection process. Each group required a certain number of the first movements:

• Group 1–zero fist movements are required.
• Group 2–single movement is require.
• Group 3–two movements are required.
• Group 4–three movements are required.

After selecting the desired group, the user chose the target letter by looking at it to get the SSVEP response. The information transfer rate when combining SSVEP and EMG was 90.9 bits/min with an average accuracy of 85.80%.

### 3.2. BCI for high-speed spelling in VR by combining eye tracking and SSVEP

**FIGURE 12:**
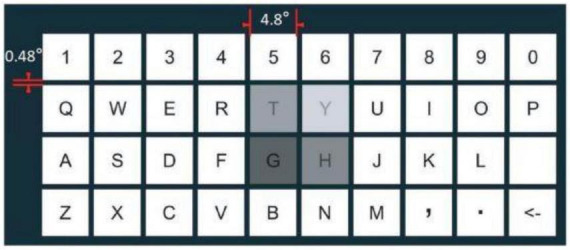
Example of the hybrid orthographic matrix in virtual reality.

Yao et al. (2018) developed a 4 × 10 orthographic matrix in virtual reality. The orthographic matrix with 40 symbols in VR was designed for two-step sequential control of eye-based block selection and SSVEP (Figure 12). Before the experiment, the subjects underwent a nine-point calibration of their eyes. During the calibration process, the subjects underwent eight iterations of fixing all 40 symbols in red (320 attempts). Then the process of selecting the symbols began. First, a block of 4 targets was selected using the gaze tracking. After selecting the desired block, the user chose the target symbol by looking at it to get the SSVEP response. This hybrid method achieved an information transfer rate of 360.7 bpm with an average accuracy of 95.2%.

Electroencephalography (EEG) indeed remains the most used BCI technique. BCI, in turn, is becoming an increasingly reliable method of experimental and clinical application due to the introduction of artificial intelligence (AI) into the brain-computer interface technology (Gutierrez-Martinez et al., 2021). Thus, in the analytical review performed on the databases of Google Scholar, PubMed, IEEE Xplore and Elsevier Science Direct, it was shown that hDL-based BCI, apparently, will help overcome a significant drawback of the EEG signal classification (Alzahab et al., 2021). Most of the studies used a Convolutional Neural Network-Recurrent Neural Network (CNN-RNN) architecture, and half of the studies used a small number of layers to achieve a compromise between network complexity and computational efficiency (Fujiwara and Ushiba, 2022). Further, the review also shows that it is necessary to use the neuroimaging method in the BCI paradigm, such as functional near-infrared spectroscopy (fNIRS), which becomes highly informative one (Gutierrez-Martinez et al., 2021). Also it is important to apply the new combinations of architectures, such as RNN and Deep Belief Network based on DBN, for a better study of the frequency and time-frequency characteristics of the recorded brain signals.

## 4. Conclusion

We have previously reviewed past research on BCI-speller based P300, MI, SSVEP and speller hybrid systems. Table 1 presents summary data, which most clearly reflects the pros and cons of the existing BCI-speller. All the non-invasive BCI-spellers presented in this review were developed with the aim of improving spelling in brain-computer systems. The use of different BCI paradigms allows for a differentiated choice, which is especially important when working with patients with lesions in different parts of the brain. It should be noted that there are major limitations in the use of BCI with neurological diseases. for the implementation of all paradigms, a sufficient level of the patient’s cognitive functions is required. This is primarily due to understanding the instructions and the ability to maintain a sufficient level of attention. For many decades, scientists have been working on increasing the speed, accuracy, and convenience of speller systems, so that they can compete with traditional methods of communication or come as close as possible to them.

**TABLE 1:** Summary of P300, MI, SSVEP spellers.

References	Subjects	Typing speed	Mean accuracy
**BCI**–**P300 paradigm**			
Farwell and Donchin, 1988	4 healthy	12 bits/min	95.0%
Jin et al., 2010	8 healthy	14,8 bits/min	92.9%
Jin et al., 2012	9 healthy	27.1 bits/min	94.8%
Acqualagna et al., 2013	9 healthy	1.4 char/min	88.4%
Akram et al., 2015	10 healthy	average time of 1.67 min per word	-
Pires et al., 2012	10 able-bodied participants, 5 participants with cerebral palsy (CP), 1 participant with Duchenne muscular dystrophy (DMD)	26.11 bits/min	89.9%
Ganin et al., 2020	9 participants with ischemic stroke in the territory of the left middle cerebral artery	average typing time per letter 38.9 s	92.1%
Korkmaz et al., 2022	10 healthy	–	93.90% for 1 flashing, 98.10% for 3 flashings and 99.81% for 15 flashings
**BCI—MI paradigm**			
Blankertz et al., 2007	2 healthy	7.6 bits/min	–
Varsehi and Firoozabadi, 2021	105 healthy	–	93.03%
**BCI—SSVEP paradigm**			
Volosyak et al., 2009	32 healthy	22.6 bits/min	92%
Sun et al., 2022	22 healthy	265.74 bits/min	76,58%
Jiang et al., 2022	10 healthy	64.35 bits/min	94,9%
Bai et al., 2023	11 healthy	28.64 bits/min	94.29%
Zhang Z. et al., 2023	7 healthy	117.05 bits/min	90,18%
Zhang J. et al., 2023	10 healthy	108.63 bits/min	94.75%
**Hybrid speller**			
Mannan et al., 2020	20 healthy	184.06 bits/min	90.35%
Zuo et al., 2020	18 healthy	–	93.94%
Zuo et al., 2020	14 healthy	53.06 bits/min	–
Lin et al., 2016	10 healthy	90.9 bits/min	85.8%
Yao et al., 2018	3 healthy	360.7 bits/min	95.2%

Despite the fact that in the process of studying a large number of works there are a small number of topics, the emphasis is on the study of EEG changes when using these technologies (Varsehi and Firoozabadi, 2021). The results achieved have demonstrated Mean Accuracy, as a rule, sufficient for practical use, reaching values of 99.8% (Korkmaz et al., 2022). However, for everyday use as a method of typing text messages, this technology is not fast enough, reaching a maximum of 360.7 bits/min (Yao et al., 2018). In this case, there is mainly a significant decrease in accuracy with an increase in the BCI speed (Sun et al., 2022). More attention needs to be paid to the graphical interface, making the design of the devices simple and user-friendly for the end consumer. Additionally, more research should be conducted directly on patients, rather than healthy subjects. At the same time, the use of a limited number of functions to control devices in the smart home concept or hospital equipment by the patient can increase the practical implementation of the technology. Increasing speller systems flashing duration, can be used as an Accuracy BCI boost (Korkmaz et al., 2022). The use of these technologies in patients, primarily with severe motor or speech disorders, on the one hand, with the improvement and simplification of the hardware part of the data, namely, with the preservation of information content (accuracy and speed) with a minimum number of recorded EEG electrodes. Thus, technologies of the brain-computer interface presented in the paper as a means of communication for people with motor disabilities serve as a good foundation for further development and research in the field of BCI-speller (Maslova and Pyatin, 2022). First of all, it concerns the direct connections between the technological solutions and targeted brain areas, which are the most effective information channels for environment interacting and communication between users based on faster, more accurate and user-friendly BCI-speller systems.

## Author contributions

All authors listed have made a substantial, direct, and intellectual contribution to the work, and approved it for publication.
